# Patterns of Phylogenetic Diversity of Subtropical Rainforest of the Great Sandy Region, Australia Indicate Long Term Climatic Refugia

**DOI:** 10.1371/journal.pone.0153565

**Published:** 2016-04-27

**Authors:** Marion G. Howard, William J. F. McDonald, Paul I. Forster, W. John Kress, David Erickson, Daniel P. Faith, Alison Shapcott

**Affiliations:** 1 Genecology Research Centre, Faculty Science Health Education, University Sunshine Coast, Maroochydore, Queensland, Australia; 2 Queensland Herbarium, Queensland Department of Science, Information Technology and Innovation, Brisbane Botanic Gardens, Toowong, Queensland, Australia; 3 National Museum of Natural History, Smithsonian Institution, Washington, DC, United States of America; 4 Australian Museum, Sydney, New South Wales, Australia; Chinese Academy of Forestry, CHINA

## Abstract

Australia’s Great Sandy Region is of international significance containing two World Heritage areas and patches of rainforest growing on white sand. Previous broad-scale analysis found the Great Sandy biogeographic subregion contained a significantly more phylogenetically even subset of species than expected by chance contrasting with rainforest on white sand in Peru. This study aimed to test the patterns of rainforest diversity and relatedness at a finer scale and to investigate why we may find different patterns of phylogenetic evenness compared with rainforests on white sands in other parts of the world. This study focussed on rainforest sites within the Great Sandy and surrounding areas in South East Queensland (SEQ), Australia. We undertook field collections, expanded our three-marker DNA barcode library of SEQ rainforest plants and updated the phylogeny to 95% of the SEQ rainforest flora. We sampled species composition of rainforest in fixed area plots from 100 sites. We calculated phylogenetic diversity (PD) measures as well as species richness (SR) for each rainforest community. These combined with site variables such as geology, were used to evaluate patterns and relatedness. We found that many rainforest communities in the Great Sandy area were significantly phylogenetically even at the individual site level consistent with a broader subregion analysis. Sites from adjacent areas were either not significant or were significantly phylogenetically clustered. Some results in the neighbouring areas were consistent with historic range expansions. In contrast with expectations, sites located on the oldest substrates had significantly lower phylogenetic diversity (PD). Fraser Island was once connected to mainland Australia, our results are consistent with a region geologically old enough to have continuously supported rainforest in refugia. The interface of tropical and temperate floras in part also explains the significant phylogenetic evenness and higher than expected phylogenetic diversity.

## Introduction

The increasing rate of biodiversity loss and pressure from anthropogenic activity in highly biodiverse rainforest communities has been documented globally [1–4]. Australian rainforests contain a high proportion of the continents terrestrial biodiversity [5]. The effective conservation of maximum biological and genetic diversity can be aided by the conservation of phylogenetic diversity (PD) or evolutionary variation [6–8].

Australian rainforests are an assemblage of relict and divergent Gondwanan elements and more recent immigrant lineages from Indo-Malesia, which exist in an archipelago of isolated patches within a matrix of sclerophyll communities [9, 10]. Rainforest contractions and expansions during the Quaternary, caused by glacial-interglacial oscillations, restricted rainforest to refugial areas of suitable climate and resulted in rainforest occupying less than 1% of the total surface area of Australia, located mostly in Queensland [5,11]. Australian subtropical rainforests have retained significant numbers of Gondwanan lineages indicating that some areas may be considered long-term rainforest refugia [12]. Subtropical rainforests of South East Queensland (SEQ) are highly fragmented and subject to intense levels of anthropogenic pressure [13]. SEQ subtropical rainforest has been identified as a distinctive region at a junction between tropical and temperate climatic zones (32–25°S), in an area often referred to as the McPherson-Macleay overlap [14, 5]. Heads [12] identified the McPherson-Macleay overlap as a global basal centre of endemism due to the presence of species that are basal to more globally widespread groups. Weber *et al*. [11] found that the area is host to large numbers of narrow range endemics consistent with climatic refugia.

The Great Sandy Region (GSR) is internationally significant containing two World heritage listed areas (Fraser Island and Great Sandy) and a biosphere reserve. The GSR has been identified as at the confluence of tropical and temperate influences, hence is an important region to observe climate change impacts. The area is notable for the presence of species of both tropical and Gondwanan influence such as *Agathis robusta* [15].

It has been argued that the ecology of islands or patches, or habitat filtering, may exhibit control of species distribution and diversity more than the area of the patch [16]. Thus rainforest patches may display patterns of diversity related to topographic and habitat heterogeneity [14]. Shapcott et al. [13] made broad biodiversity assessments of SE Qld rainforest within the SE Qld bioregion at the subregion level using phylogenetic diversity (PD) measures and found that rainforest diversity was not correlated with the area of rainforest present in SEQ biogeographic subregions. Phylogenetic diversity (PD) is a measure of biodiversity that incorporates evolutionary relationships among taxa [6]. Costion et al. [8] found a strong correlation between phylogenetic diversity (PD) and climatic refugia in tropical Australian rainforest using DNA barcoding. Communities containing phylogenetically clustered taxa, contain species more closely related than expected by chance resulting from dispersal limitations or habitat specialisations [17, 18]. Whereas, communities exhibiting phylogenetic evenness, contains species less closely related then expected by chance [19]. Phylogenetic evenness has been found in older more stable refugial habitats [18, 19] which may result from competitive exclusion, limiting similarity on conserved niches or filtering of convergent characters [11,13]. Studies of rainforests have found patterns of both phylogenetic evenness and clustering [13, 18, 19].

Plant community structure is the product of many factors including, climate, disturbance, topography and geological substrate [20,21]. Australia has been mapped into Bioregions which are broad-scale biogeographical units whose limits are naturally defined by common geology, topographic and biological features [22] and have been further classified into subregions based on a finer scale commonality of geology and landform but also includes broad vegetation types (IBRA) (www.environment.gov.au) [23]. Geology may contribute to the existence of habitats that exhibit refugial characteristics, such as endemism, due to geographic and edaphic discontinuity and isolation [21]. Therefore, it may be hypothesised that geological age and type may correlate significantly with PD. The Great Sandy Region (GSR) consists of the sand masses and sandstone hills of World Heritage listed Fraser Island and the Cooloola sand mass, characterised by perched, barrage and window dune lakes, and the riverine plains of the upper Noosa Catchment (www.environment.gov.au/land/nrs), [24,25]. White sandy substrates are thought to be geologically younger than other geology types and of low nutrient levels thus would be predicted to contain phylogenetically clustered communities. Fine and Kembel [26] found that rainforest sites in the white sand forests of Peru were significantly phylogenetically clustered. In contrast Shapcott et al. [13] found that the SEQ subregion which includes the Great Sandy Region (GSR) and Fraser Island, exhibited significant phylogenetic evenness however it is unknown if this pattern is expressed across the region or focused on rainforest communities at specific locations.

This study builds on the preliminary SEQ rainforest DNA barcoding study by Shapcott et al. [13] with the expansion of the DNA barcode library to obtain close to 100% representation of species. The study then aimed investigate more closely patterns found in the GSR and surrounds, specifically to:

1. Conduct a finer scale analysis of phylogenetic diversity within the GSR using plot data to determine if the phylogenetic diversity found by Shapcott et al. [13] at the subregion level is also found at the site level.
2. Examine the local patterns and relationships of species composition and PD among rainforest sites within the GSR and surrounds, particularly to the north, to define the local geographic boundaries of diversity and significant phylogenetic evenness or clustering.
3. Determine if geological age and type are correlated with PD or evenness in the GSR and surrounds.

## Materials and Methods

### Experimental design and field methods

**Fig 1:**
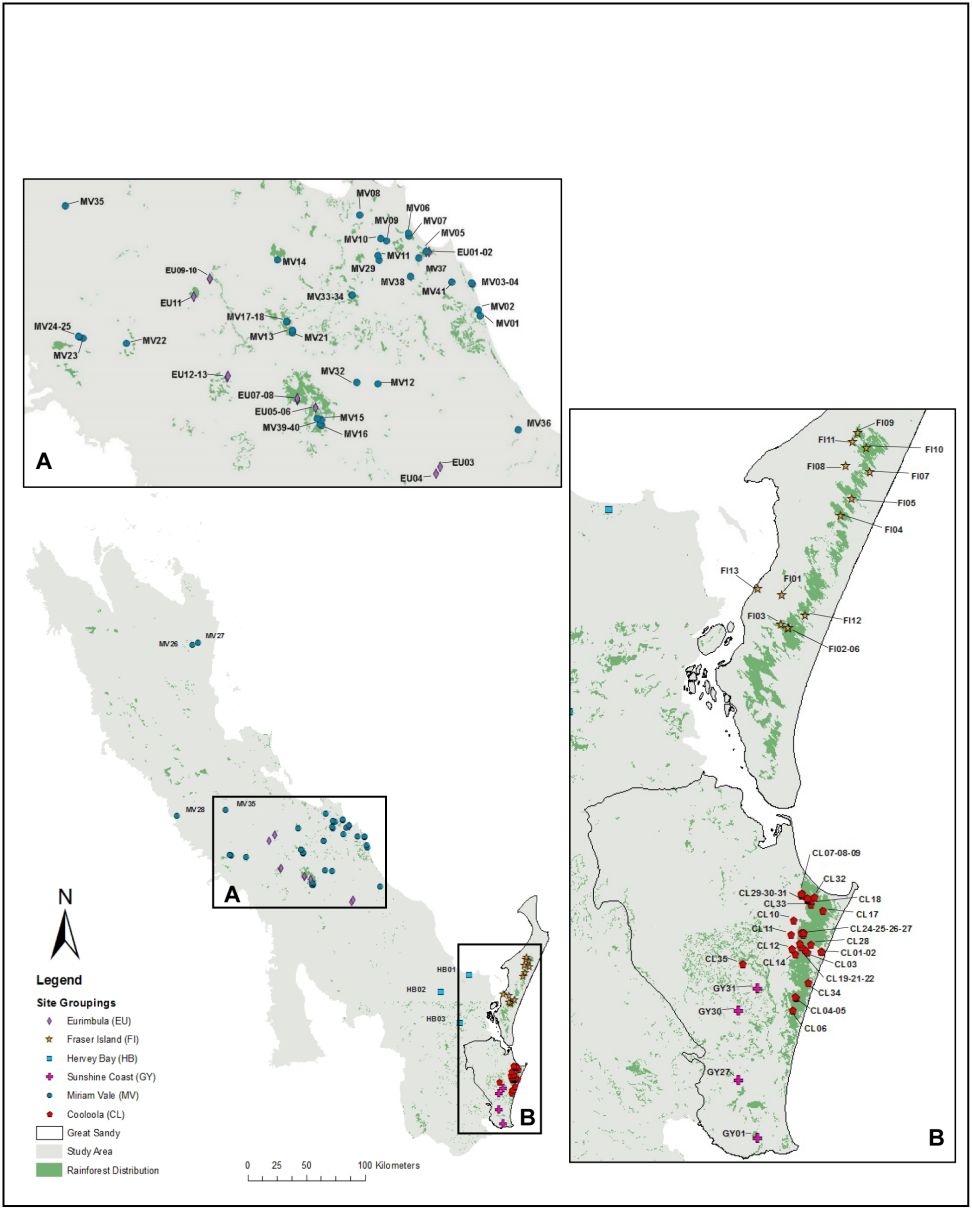
Map indicating the extent of the study area with expanded sections to show the location of the 100 study sites within broad geographic groupings (A) Eurimbula (EU) and Miriam Vale (MV): B) The Great Sandy Region. Fraser Island (FI), Cooloola (CL) and Sunshine Coast (GY). The extent and distribution of rainforest within this landscape is illustrated with solid green shading.

Rainforest currently exists as small patches within a matrix of drier vegetation types, the study area focused on rainforest containing sites within the GSR as well as the northern extent of the McPherson-Macleay overlap (Fig 1). This complements previous studies in the rainforest to the south in New South Wales and around the Boarder Ranges with known centres of diversity [13]. We prepared a list of 162 species targeted for collection in order to complete the collection of all SEQ rainforest plant species for the DNA barcode library. Queensland Herbarium (BRI) ‘Herbrecs’ database was used to obtain locations assisting with field searches. Field permits were issued by the Queensland Environmental Protection Agency. Samples of at least one herbarium voucher specimen and one DNA voucher preserved in silica gel [13] were lodged at the Queensland Herbarium. A duplicate DNA voucher for each specimen was kept at the University of the Sunshine Coast (USC). All specimen identifications were confirmed by Queensland Herbarium staff.

The rainforest distribution was sampled from 100 sites within the GRS as well as from adjacent areas particularly the coastal areas to the north to investigate local patterns of diversity and composition (Fig 1). The flora from each site was sampled within a fixed area plot (0.1 ha, W.J.F. McDonald, unpub. data) as per Butler et al. [27] consistent with vegetation survey methods used by the CORVEG database [28] which are selected to be representative of the vegetation type and used for vegetation mapping in Queensland. Average rainfall data within the study area were obtained from the Bureau of Meteorology (http://www.bom.gov.au/climate/data/stations/) [29].

### Laboratory Methods

Laboratory methods were consistent with Shapcott et al. [13]. Polymerase chain reactions (PCR) were performed for each sample for each of the three markers; *mat*K, *rbc*L and *psb*A*-trn*H. The PCRs were performed on a Kyratec SuperCycler Trinity PCR cycler. The success of the PCR was tested by electrophoresis on 1.5% agarose gel with ethidium bromide and viewed under UV light on a Molecular Imager^®^ Gel Doc XR plus with Image Lab^™^ Software Version 5.0. The PCR products were purified using ExoSAP-IT^®^ (USB^®^). The forward and reverse cycle sequencing reactions were performed using a 12 μL sequencing mix consisting of 5 X sequencing buffer, 0.5 μL of BigDye^®^, 100 μM M13 primer (forward or reverse) and 4 μL of purified PCR product. Cycling conditions were the same for all markers. The final product was purified through a Sephadex column and sequenced in an ABI 3500 Genetic Analyser.

### Phylogenetic tree construction

Following a similar method as outlined by Shapcott et al. [13], sequence output was edited in Geneious^®^ v6.1.7and contigs were made from forward and reverse sequences. The new sequences were added to the existing SEQ library [13]. A consensus alignment for the *rbc*L gene was performed with ClustalW [30] and then manually aligned with the larger SEQ *rbc*L consensus alignment. The program MUSCLE [31] was used for *mat*K and manually realigned. For *psb*A*-trn*H, sequences were exported to SATé v2.2.7 [32] for alignment, which used MAFFT [33], MUSCLE [31] and FASTTREE [34]. Preliminary phylogenetic trees were generated for each marker separately, *rbc*L, *mat*K and *psb*A*-trn*H, and used to check for obviously out of place samples which were either corrected, re-analysed or removed. Nucleotide sequences for the three markers were then concatenated for form a 3-marker alignment.

Phylogenetic reconstruction followed a similar method outlined by Shapcott et al. [13]. A constraint tree for phylogenetic analysis was generated using the updated list of 810 rainforest taxa in Phylomatic (v3) [35] which applied the APGIII base tree [36] and the R20120829 phylomatic tree for plants (http://phylodiversity.net/phylomatic/). The terminal branches of the output tree were collapsed to Family using the program Mesquite (http://mesquiteproject.wikispaces.com/) [37]. The constraint tree, 3-marker alignment and an *rbcL*, *matK*, *psbA-trnH* data partition file were uploaded to The CIPRES Science Gateway V.3.3 (http://www.phylo.org), [38] RAxML-HP2 on XSEDE was used to a phylogenetic tree which included branch lengths and a mixed partition model [39] The best tree out of a total of 64 randomisations was used for final analyses. PATHd8 program [40] was used to date the final best tree. This was done by assigning the age of nine Orders to major branch nodes representing major angiosperm evolution. The nine Orders were similar to those used by Shapcott et al. [13] with some variation due a difference in species included in the phylogenies (S1 Table). We also assigned a date for angiosperms of 250 Ma as applied previously by Magallón and Castillo [41] (S1 Table). The final dated SEQ rainforest tree was then used for PD estimations.

### Community data analysis methods

Species lists from each of the 100 sites were used to create community files using the species barcode identifiers for each study site. Only the species that were included in the final DNA barcoded phylogeny were included in the species community list generated for each study site. The dated SEQ rainforest phylogeny created above, the complete SEQ rainforest community file, and the individual site community files were used to calculate site-level phylogenetic diversity (PD) [6] and species richness (SR) using the PICANTE package in R [7, 42]. We plotted PD against SR to investigate this relationship and also plotted the fraction of GSR species out of the total phylogeny against the fraction of GSR PD out of the total SEQ PD and derived a power curve [43], we also plotted log PD against log SR and fitted a regression. The mean phylogenetic distance (MPD) and mean nearest taxon distance (MNTD) [17] were also calculated for each site, as well as for the SEQ region, using the PICANTE package in R [7, 42]. PD, MPD and MNTD for each site were tested against a randomised null model using the whole SEQ taxa to see if they deviated significantly from random chance PICANTE calculates a standardized size effect (ses) and a probability -1 times the ses for MPD gives the net relatedness index (NRI) and for MNTD the nearest taxon index (NTI) [17, 42].

To further investigate the patterns and relatedness among the site communities and to evaluate the dissimilarity in site species composition, pairwise dissimilarity matrices were calculated based on species presence/absence data using the Bray-Curtis metric generated in the Primer 6.1.5 program [44]. In addition, a phylogenetic dissimilarity (*Dpw*) matrix based on MPD, and mean nearest phylogenetic neighbour distance (*Dnn*) between communities based on MNTD [17] were calculated in PICANTE [7, 42]. A dissimilarity matrix among sites was calculated based on PD using Fast Unifrac (UnifracPD; http://unifrac.colorado.edu/) [13, 45]. These distance matrices were used to undertake non-metric multidimensional scaling (NMDS) analysis in the Primer 6.5.1 program [44] to investigate the relationships among the sites. To test for significant correlations among the diversity measures (SR, PD, MPD, MNTD), pairwise dissimilarity matrices were tested using a Mantel test equivalent (RELATE function) in the Primer 6.1.5 program [44].

We explored patterns for six arbitrary geographic groupings: Cooloola (CL); Eurimbula (EU); Fraser Island (FI); Sunshine Coast (GY); Hervey Bay (HB) and Miriam Vale (MV). Sites were grouped according to geological age and geology type according to the Geological Survey of Queensland [46] and mapped in ArcGIS 10.2 in relation to rainforest patches and surface geology [47]. Rainforest distribution layers were obtained from the regional ecosystem post-clearing data sets of the Queensland Herbarium [48]. Geology distribution layers were obtained from the Geological Survey of Queensland [46]. To investigate the relationship between significant phylogenetic evenness or clumping (NRI) and geology and rainforest distribution these sites were indicated on the maps. One-way ANOVA was undertaken to test if there were significant differences between groupings of geographic area, geological age and geological type and the PD of each site. A Tukey HSD post hoc test was conducted to identify groups where there was significant difference for each analysis undertaken using SPSS [49]. To assist in the investigation of the relationships between the sites grouped into geographic areas, the iTOL program (http://itol.embl.de/) was used to generate a circular phylogenetic tree with the composition of the different sites indicated in order to see phylogenetic patterns [50].

## Results

### Diversity

**Fig 2:**
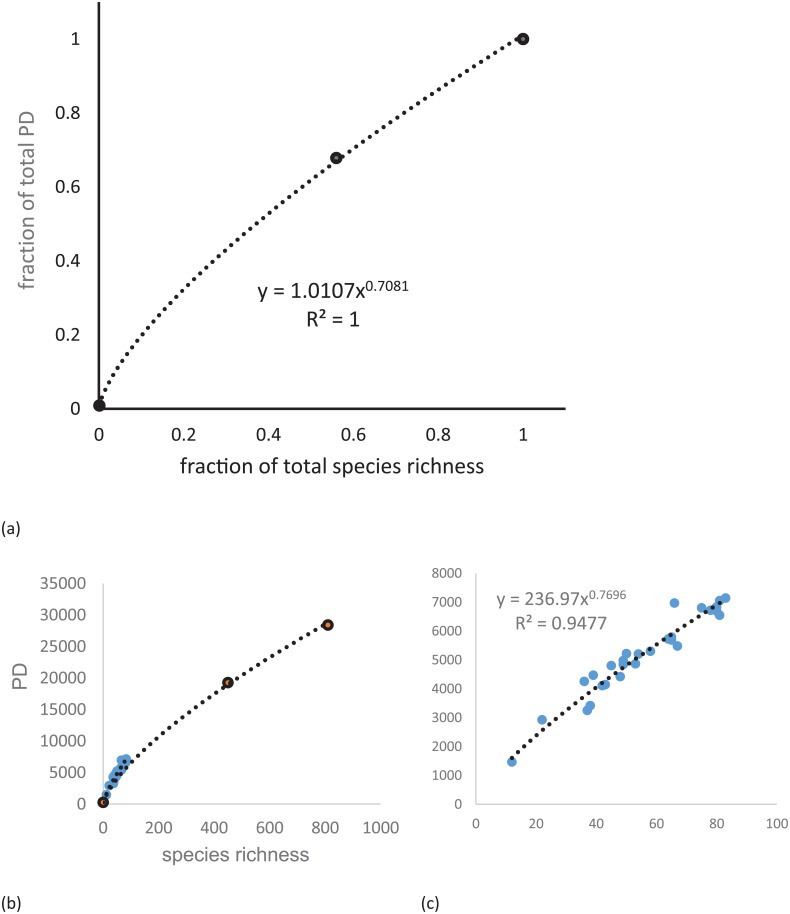
The PD—species richness (SR) relationship in the study area. (a) The power curve plotting the fraction PD vs fraction of SEQ rainforest species generated using the entire SEQ phylogeny the equation for the curve and R^2^ value is given; (b) PD vs SR showing the power curve for SEQ and the curve for the 100 study sites in relation indicating the greater slope; (c) enlarged Site PD vs SR for the 100 study sites showing the trend line and its equation.

**Fig 3:**
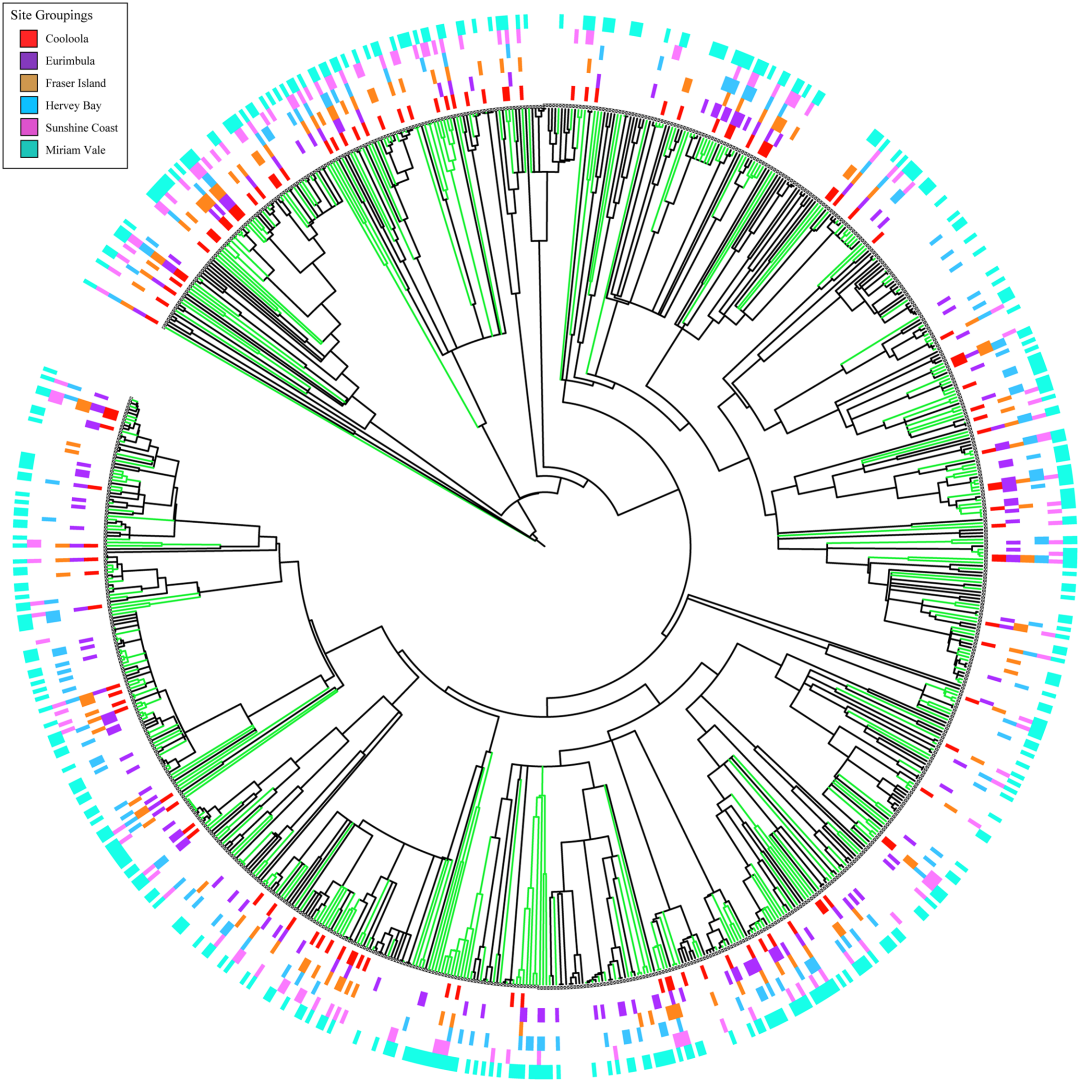
Graphical representation showing species within the study area (green branches) within the total SEQ rooted and dated constrained phylogenetic tree constructed from the 3-gene DNA barcode data. The species within each geographical group: Miriam Vale (MV), Eurimbula (EU), Cooloola (CL), Fraser Island (FI) Sunshine Coast (GY) are shown by colour bars at branch tips. The colours are indicated.

The expanded SEQ phylogeny was constructed based on 810 species representing 95% of SEQ rainforest plants. The Great Sandy and neighbouring areas used in this study contain a subset of 56% of the species composition of the total SEQ phylogeny (457/810 species). The PD for the whole of the SEQ community phylogeny was 28396.36 and for the Great Sandy study area total PD was 19260.87 (67.8% of the total; Table 1). We found the PD-species relationship fits a power curve with a z value of 0.708 (Fig 2). Across the 100 study sites PD was strongly correlated with SR (R^2^ = 0.948; Fig 2) and the study site PD was consistently slightly higher against SR than observed for SEQ generally (Fig 2). The species composition of the total study area is sampled relatively evenly from across the entire SEQ total species pool (Fig 3). However, the more northern sites of the Miriam Vale (MV) and Eurimbula (EU) groups included taxa that were absent from the more southern sites of Cooloola (CL), Fraser Island (FI) and the Sunshine Coast (GY; Fig 3), suggesting greater influence of northern rainforest taxa.

**Table 1:** Summary of the mean values of the SEQ subtropical Rainforest diversity and geological characteristics of the Great Sandy subregion and neighbouring subregions grouped by geographic community.

Community	No. of Sites	SR	PD	MPD	NRI	Significant NRI (%)	MNTD	NTI
**Cooloola (CL)**	30	111	4133.54^EU,GY,MV^	373.76	-1.60	23.2	136.32	0.78
**Eurimbula (EU)**	13	142	2350.96^CL,FI,GY,HB,MV^	331.21	0.84	0.0	191.86	0.25
**Fraser Island (FI)**	13	113	4952.51^EU,MV^	377.41	^E^*-2.19	69.2	140.65	0.24
**Sunshine Coast (GY)**	4	131	6084.83^CL,EU^	375.90	^E^*-2.5	100.0	107.97	1.01
**Hervey Bay (HB)**	3	132	6121.14^EU^	352.89	-0.06	0.0	121.70	0.17
**Miriam Vale (MV)**	37	372	6462.74^CL,EU,FI^	356.02	-0.30	16.2	113.51	0.32
**F**			26.281**					
**TOTAL**	100		19260.87	361.60	89.88	26.0	134.09	-0.09

SR, species richness; PD, phylogenetic diversity; MPD, mean PD of taxa within the community; MNTD, mean nearest taxon distance within the community; NRI, net relatedness index within the community; NTI the nearest taxon index within the community.

Significant values are indicated (* p<0.05).

F values of One-way ANOVA are given (** p<0.001).

The results of Tukeys HSD post hoc test are indicated by superscript: CL, EU, FI, GY, HB, MV.

^E^ indicates significant taxonomic evenness.

**Fig 4:**
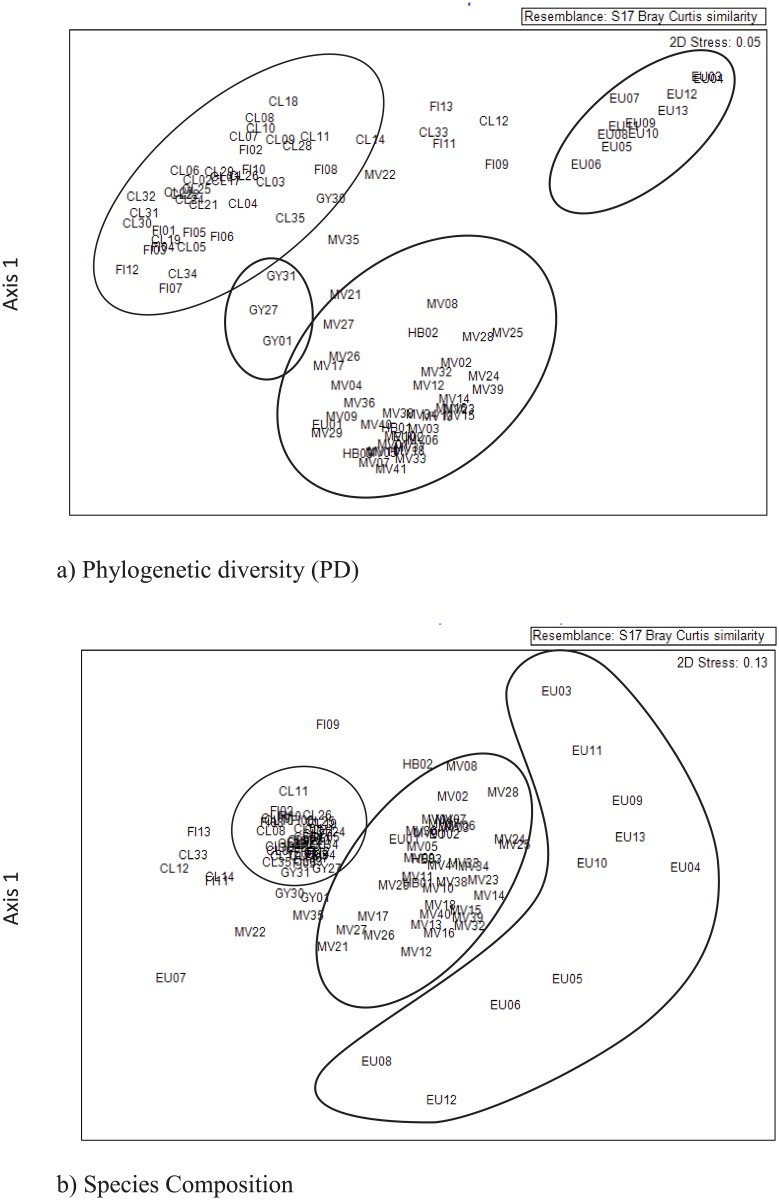
Map of the study area showing which sample sites are significantly even or clustered in relation to the extent of rainforest (shown in green). The boundaries and codes of subregions within the study area are indicated showing their relation to the location of Great Sandy Region (12.9).

There was a significant difference (F = 26.281, p <0.001) in the PD among geographical areas. The sites in Miriam Vale (MV) group had the highest diversity and contained the sites with highest species richness (SR = 372) and phylogenetic diversity (PD = 6462.74; Table 1), reflecting the contribution of tropic and temperate influences. Fraser Island sites did not have the lowest diversity as might be expected for an island. Rather the sites in the Eurimbula (EU) area had significantly lower PD compared to all other sites (p<0.001; Table 1). The lowest diversity was found at the Eurimbula site EU03 (SR = 6; PD = 1026.23; Fig 4). The site with the highest diversity on Fraser Island (FI) was FI07 (SR = 83; PD = 7145.82; Table 2).

**Table 2:** Summary of the SEQ subtropical Rainforest diversity for individual study sites in the Great Sandy subregion and neighbouring subregions which had significant (p<0.05) NRI or NTI values.

Community	Site	SR	PD	MPD	NRI	MNTD	NTI
**Cooloola**	CL34	54	5203.66	375.59	^E^*-2.17	130.22	0.27
	CL22	53	4865.68	374.36	^E^*-2.03	117.91	1.21
	CL21	49	4975.73	374.78	^E^*-1.97	142.02	-0.17
	CL05	45	4799.89	382.61	^E^*-2.51	151.13	-0.36
	CL04	43	4142.28	378.28	^E^*-2.09	127.53	1.17
	CL24	43	4142.28	378.28	^E^*-2.09	105	*1.97
	CL18	38	3420.26	376.89	-1.91	114.25	*2.31
	CL28	37	3250.86	369.48	-1.28	9.46	*2.60
	CL14	22	2924.74	389.87	^E^*-2.16	201.65	-0.41
**Eurimbulah**	EU08	12	1467.56	345.33	0.33	128.43	*2.82
**Sunshine Coast**	GY27	80	6833.36	375.83	^E^*-2.63	105.65	0.71
	GY01	78	6719.92	379.75	^E^*-2.97	112.17	0.26
	GY31	67	5484.26	372.00	^E^*-2.07	101.46	1.73
	GY30	58	5301.77	376.04	^E^*-2.33	112.58	1.36
**Fraser Island**	FI07	83	7145.82	374.23	^E^*-2.50	115.70	-0.31
	FI01	80	6692.94	375.19	^E^*-2.61	110.56	0.29
	FI12	81	6548.68	370.88	^E^*-2.12	104.96	0.74
	FI04	65	5792.19	375.93	^E^*-2.36	122.94	0.13
	FI03	64	5723.77	374.78	^E^*-2.27	121.02	0.34
	FI06	49	4845.96	381.09	^E^*-2.55	133.68	0.37
	FI08	39	4470.98	394.96	^E^*-3.32	170.53	-0.98
	FI10	48	4423.67	379.79	^E^*-2.40	123.29	1.07
	FI02	42	4107.26	377.30	^E^*-2.02	129.82	1.10
**Miriam Vale**	MV12	81	7055.18	373.60	^E^*-2.34	111.35	0.21
	MV27	66	6972.06	379.50	^E^*-2.68	158.86	*-2.69
	MV29	75	6808.37	376.37	^E^*-2.53	125.17	-0.72
	MV02	65	5687.32	332.32	^C^*2.05	112.40	1.01
	MV21	50	5217.03	382.36	^E^*-2.64	134.34	0.28
	MV35	36	4259.49	390.36	^E^*-2.79	177.50	-0.96

^E^ indicates significant taxonomic evenness (less closely related than random).

^C^ indicates significant taxonomic clustering (more closely related than random).

SR, species richness; PD, phylogenetic diversity; MPD, mean PD of taxa at each site; MNTD, mean nearest taxon distance within each site; NRI, net relatedness index within each site;

NTI, nearest taxon index within each site, significant values are indicated (* p<0.05).

### Patterns

**Fig 5:**
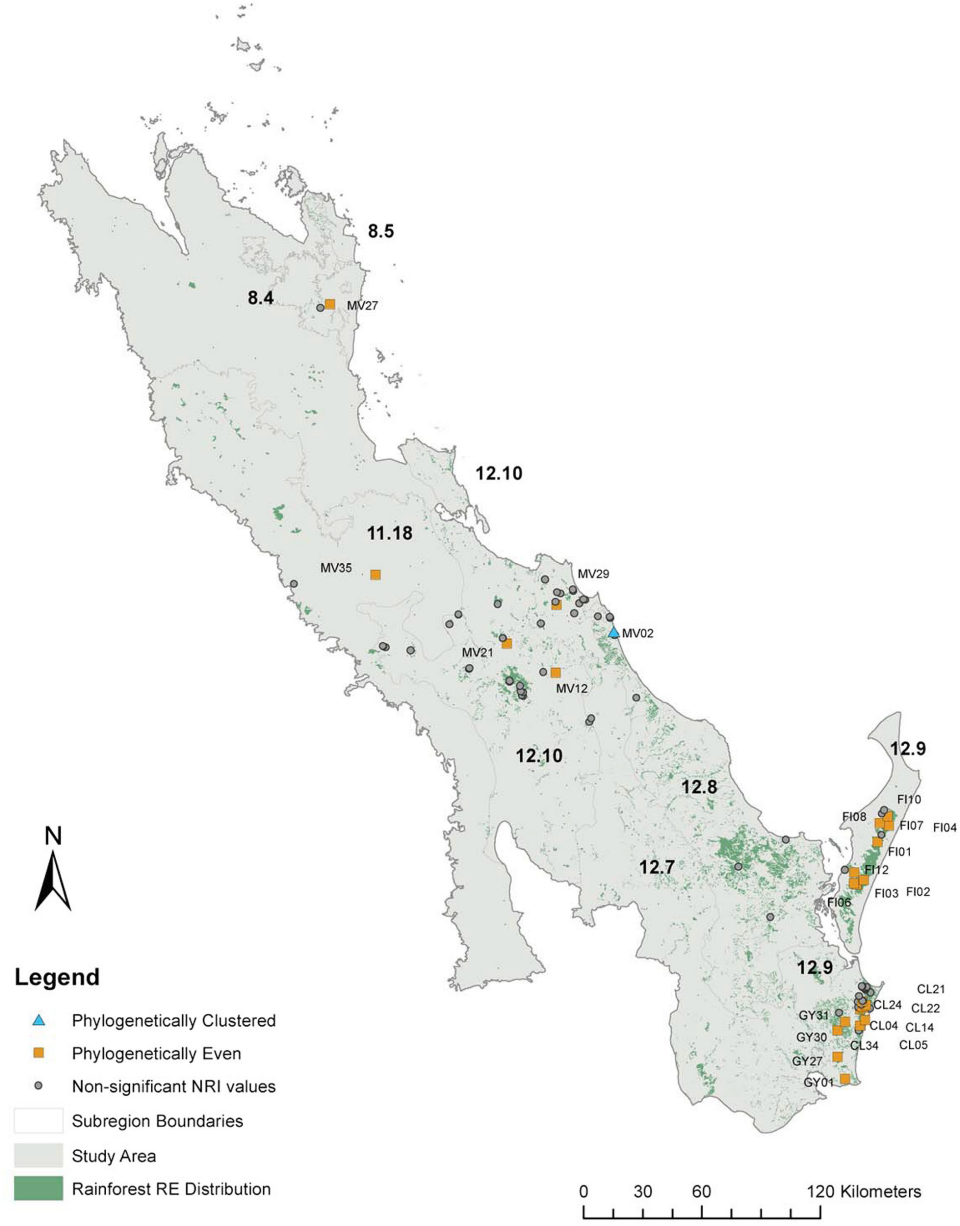
Non-metric multidimensional scaling (NMDS) analysis comparing relationships among the study sites for SEQ subtropical rainforest taxa. (a) PD using the Unifrac dissimilarity matrix. (b) Species composition using Bray-Curtis dissimilarity matrix.

Contrary to expectations, the PD of the Sunshine Coast (GY) sites was significantly higher than the PD of the neighbouring Cooloola (CL) sites (p = 0.05). The NMDS for PD distance illustrates this difference in phylogenetic composition for the Sunshine Coast (GY) sites which are grouped in the centre of the ordination (Fig 5). The NMDS of phylogenetic diversity distance (PD) and species composition distance illustrates that there are three distinct groupings (Fig 5). Fraser Island (FI) and Cooloola (CL) sites group together, Miriam Vale (MV) and Hervey Bay (HB) sites group together while the Eurimbula sites group is distinct from the others (Fig 5). Rainfall patterns in the study area indicate that southern and coastal groups have higher average rainfall than northern groups (Table 1).

Overall the Fraser Island (FI) and the Sunshine Coast (GY) sites were significantly phylogenetically even (NRI; Table 2; Fig 4) whereas Cooloola (CL) sites overall were not. All sites in the Sunshine Coast (GY) group were significantly phylogenetically even. Nine out of 13 (77%) Fraser Island (FI) sites were significantly phylogenetically even and seven out of 30 sites (23%) in Cooloola (CL) were significantly even with the rest being non-significant. These results are thus consistent with the broad analysis of Shapcott et al. [13] and show that evenness is observed at the small sample site as well as the broad whole of subregion level. The results also provide finer scale detail of where the patterns are found (Figs 1 and 4).

Outside the GSR to the north, few sites were non-random (Table 2; Fig 4) except for six individual sites in the Miriam Vale area. Only six sites (16.2%) were significantly phylogenetically even (NRI; Table 1; Fig 4). Only one site in the study area was significantly phylogenetically clustered, the coastal Miriam Vale site MV02, indicating that the species in this site were more closely related than random (Table 2; Fig 4). This site was located at Deepwater National Park in endangered sandy beach ridge littoral rainforest isolated from other rainforest communities by sclerophyllous open Eucalypt and low open *Banksia aemula* woodlands [50]. Sites in the Cooloola (CL), Fraser Island (FI) and the Sunshine Coast (GY) groups of the Great Sandy had high MPD scores indicating that taxa are more phylogenetically different within the sites than other groups (Table 2). The site CL24 in the Cooloola (CL) group of the Great Sandy and Miriam Vale site MV27 were significantly taxonomically distinct (NTI) (Table 2; Fig 4).

### Geology and Rainfall

**Fig 6:**
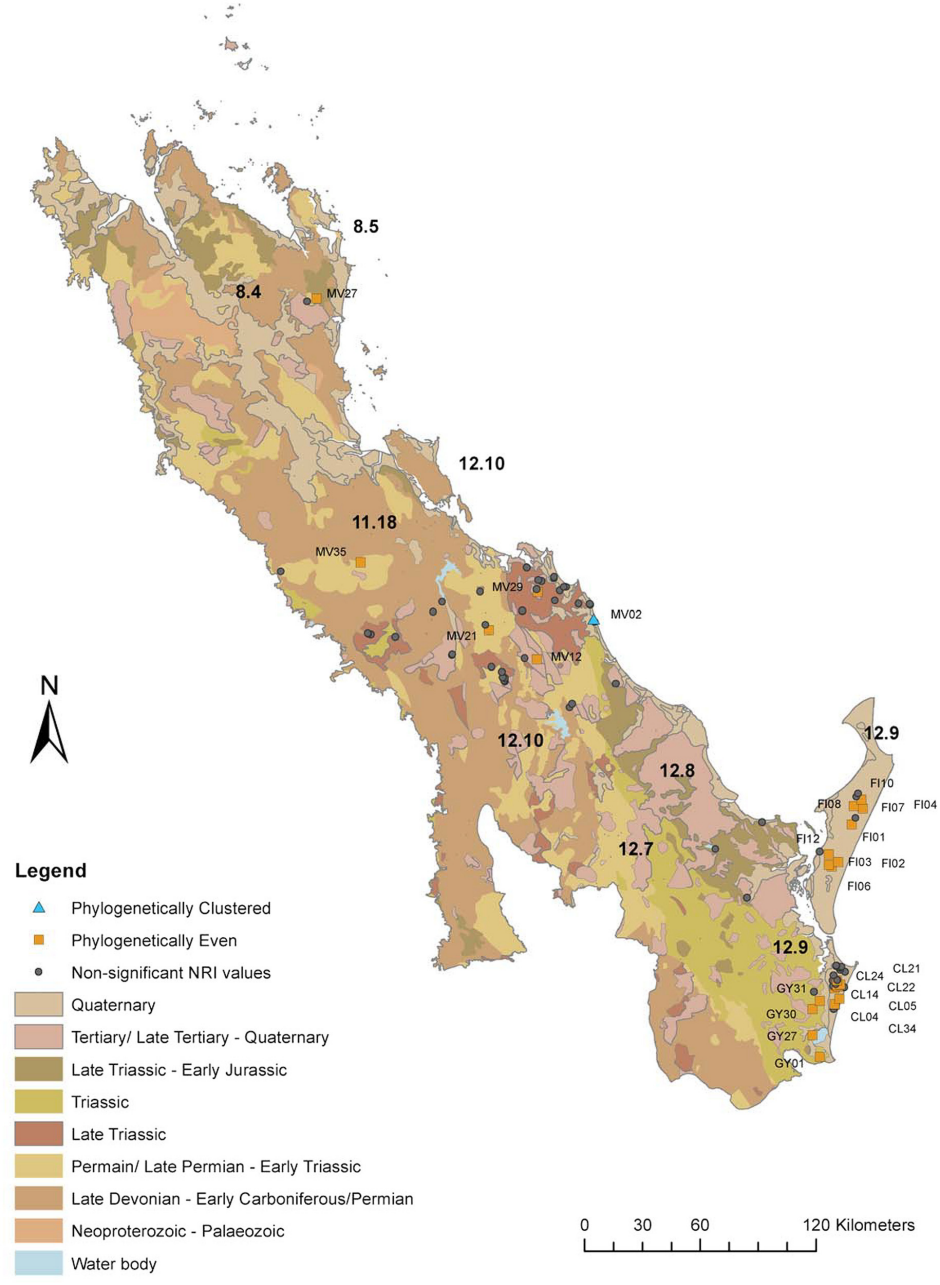
Map of the study area showing which sample sites are significantly even or clustered in relation to the geological age of substrate. The boundaries and codes of subregions within the study area are indicated showing their relation to the location of Great Sandy Region (12.9).

When grouped according to geological age (F = 3.982, <0.001) and geology type (F = 4.957, p<0.001) sites located on geology from the Metavolcanic of the Late Devonian to Permian age between 416–299 Ma from the Burnett Curtis Hills and Ranges (12.10) subregion had significantly lower phylogenetic diversity (PD; p<0.05). These are the oldest geological formations within the study area (Fig 6). The Cooloola (CL), Fraser Island (FI) and Sunshine Coast (GY) sites mainly found on Quaternary quartz sand accounted for 55% of sites within the study area (Table 1; Fig 6). However, the PD of these sites was not significantly different to other geographic areas on older or different geologies (p>0.05). The coastal sites of Fraser Island (FI), Cooloola (CL) and Byfield (MV27) areas had a high mean annual rainfall (>1500 mm) while a drier region was typical of more northern and inland sites (Table 3).

**Table 3:** The average rainfall of geographical communities in the study area.

Location of Bureau station	Subregion	Mean annualrainfall (mm)
**Mt Alma**	11.18	819.6
**Kolonga**	12.7	863.0
**Eurimbula**	12.10	996.9
**Hervey Bay**	12.8	1092.9
**Miriam Vale**	12.10	1142.5
**Boreen Point**	12.9	1364.0
**Cooloola**	12.9	1487.6
**Bulburin**	12.10	1490.4
**Fraser Island**	12.9	1590.9
**Byfield**	8.5	1683.6

## Discussion

### Diversity

Morlon et al. [43] described how PD increases with an increasing number of species randomly sampled from a given phylogeny and supported a power curve relationship between PD and number of species. In our study, we can show a very good fit for a power relationship and further that our sites have a higher PD than expected for SEQ taxa (Fig 2). This study found that while evenly distributed across the phylogeny, some species that are phylogenetically distinct, i.e. with long phylogenetic branch lengths were in Miriam Vale (MV) and Eurimbula (EU) communities but were absent from more southern Fraser Island (FI), Cooloola (CL) and the Sunshine Coast (GY). This is consistent with a higher proportion of tropical immigrant taxa such as *Hypserpa* and *Stephania* genera of the pantropic family Menispermaceae and *Drypetes* genus from the tropical family Putranjivaceae in the northern Miriam Vale (MV) sites which were absent from the less phylogenetically diverse southern sites. These results are consistent with Shapcott et al. [13] and supports the theory of temperate and tropical convergence of the McPherson-Macleay overlap [20].

The theory of Island Biogeography proposes that low species richness is related to isolation [51]. Fraser Island is geographically less isolated than oceanic islands. Fossil evidence suggests it was incorporated in the mainland during the last Pleistocene glacial maxima ~20 000 kyr when the shore line was 30–70 km east of its current position [24, 51]. The Fraser Island (FI) group was found to have lower SR than northern mainland groups but SR was similar to the neighbouring mainland Cooloola (CL).

### Patterns

In Australia, rainforest distribution and type is determined largely by water availability, PD and SR were significantly related to rainfall [20, 52]. Stable moist patches considered to be refugial areas [8] have been documented to have high levels of phylogenetic evenness (NRI) while phylogenetic clustering is more common in seasonally dry regions [13,18,52]. Sites in the Fraser Island (FI) and Sunshine Coast (GY) groups with high mean annual rainfall were significantly phylogenetically even indicative of refugial areas. Sites from the Miriam Vale (MV) and Eurimbula (EU) groups in the drier northern area (Table 1) were not significant. This is consistent with the hypotheses of high rainfall and water availability as predictors of phylogenetic diversity patterns [8,18].

Phylogenetic evenness may also be attributed to filtering of convergent characters or competitive exclusion of similar species [19]. The significantly phylogenetically even sites identified in this study correspond with areas that Weber et al. [11] recognised as areas of high endemicity indicative of climatic refugia. A study by Butler et al. [27] found large seeded and larger plant size of species in higher rainfall sites such as Fraser Island reflects evolutionary convergence of species for persistence rather than recolonisation. The Cooloola (CL) group contained sites that were significantly phylogenetically even but northern sites in this group were not. This may be consistent with a loss of diversity due to habitat filtering over time or high endemism and sympatric speciation [52, 53]. Costion et al. [8] postulated that areas of lower than expected PD correspond with refugial areas; however, the results of this study are not consistent with this hypothesis. For example, we found that the Eurimbula (EU) sites, which had the lowest PD were more genetically similar than random and contained a high proportion of congeneric species (NTI). The Miriam Vale (MV) group had five sites that were significantly phylogenetically even but these were not among those with the highest PD.

Phylogenetic clustering is considered to be the result of dispersal limitation or habitat specialisation [17]. Erickson et al. [54] found clustering apparent in more speciose sites while evenness was correlated with species poor sites in tropical and temperate sites. These findings are supported by the results of this study which found that the Miriam Vale (MV) group which contained the sites of the highest SR in the study area also contained the only site (MV02) in the study area that was significantly phylogenetically clustered (NRI). The isolation of this site may contribute to the occurrence of congeneric species by sympatric speciation of taxa filtered into shared habitats [52]. Alternatively, this site may be an example of competition with an over representation of good competitors or dispersal limitations [18, 19].

### Geology

The geology of a region is thought to be one of the determining factors that influence biodiversity patterns and habitat heterogeneity through chemical and physical properties of the soil and water availability [55]. Nichols et al. [56] in USA and Fayolle et al. [57] in Africa found SR was correlated with geomorphological heterogeneity. In Australia more complex subtropical rainforest is observed on eutrophic soils while less fertile soils support simpler vegetation [58, 59]. The GSR was predicted to host rainforest communities that are phylogenetically clustered but this area was identified by Weber et al. [11] as a refugia based on species endemism. In contrast to the results of Fine and Kemble [26], many sites in the GSR were significantly phylogenetically even. This difference is not surprising given that the formation of the sand substrates differ greatly. The white sand in the Amazon is composed mainly of quartz from eroded sandstone sediments of Precambrian origin [60], whereas the high dune sequences of Fraser Island and Cooloola are characterised by aeolian sands deposited by longshore drift and reported to be the oldest known time sequence of soils (podsols) with profiles more than 25 m deep [61,62]. The sand masses have developed episodically during fluctuation of sea levels in the late Quaternary and are aged from Holocene (<10 000 kyr) to before the last Pleistocene interglacial period 120 000–14 000 years ago [63, 24]. The GSR sites are found on nutrient rich eroded dune floors or corridors, surrounding springs and extend short distances downstream from them [15].

Modification of land surfaces and intense geologic activity during the Tertiary are thought to have led to a mosaic of sedimentary patterns including many areas of younger and recently exposed fertile soils as well as older deeply weathered very low nutrient soil [20]. Sites from the Eurimbula (EU) area on the oldest geological formations in the study area had significantly lower PD suggesting that species are more related at a basal level and have a high incidence of closely related terminal taxa [53]. This group was also located in the drier northern region. These results suggest that a combination of poor nutrient substrate related to geological age low rainfall and isolation may have favoured particular lineages.

## Conclusions

This analysis has provided a greater understanding of the distribution of biodiversity in the subtropical rainforest estate of SEQ. A more complete phylogeny of 810 species was generated using the data from the DNA barcoded library. This study demonstrated that the overall pattern found at the broad subregion level was also present at the individual site level and that this scale of sampling was sufficient to detect significant differences among sites and regions as well as determining more fine scale regional patterns. This study identified that, at a finer scale, 40% of the rainforest sites sampled in the GSR exhibited significant phylogenetic evenness suggestive of potential rainforest refugia. The study found that the combination of rainfall, nutrient levels and geological age of the soils of Fraser Island and Cooloola are likely to have been sufficient to maintain rainforest over extended timeframes. Therefore, combined evidence from this study supports the theory that this area could have been a rainforest refugia. Communities from more northern sites of the Miriam Vale area had the highest PD and SR and the geologically oldest sites in the Eurimbula (EU) group had the lowest PD. Only one significantly phylogenetically clustered site, on sand was identified as potentially indicative of more recent colonisation. This study emphasises the importance of the Great Sandy Region for the conservation of phylogenetic variability necessary for the recolonisation of surrounding subregional landscapes in the event of disturbance especially as the result of future climate change.

## Supporting Information

S1 Table Summary of nine Orders, dates used and the reference pertaining to each (Shapcott *et al*., 2015) used to date the SEQ rainforest phylogenetic tree in the PATHd8 program. Where MRCA is the most recent common ancestor of the two taxa that span the clade being dated on the tree.
(DOCX)

S2 Table Summary Table of details for all new additions to the SEQld rainforest barcode phylogeny. The species name, Genbank accession codes for each of the three DNA barcode markers used as well as the Queensland Herbarium (BRI) collector name or number which is used by BRI as specimen identifier as well as the BRI accession number where available at time of submission.
(DOCX)
