# Supplementary material for: Patterns of Phylogenetic Diversity of Subtropical Rainforest of the Great Sandy Region, Australia Indicate Long Term Climatic Refugia
Source: PLoS One. 2016 Apr 27;11(4):e0153565. doi: 10.1371/journal.pone.0153565 (PMC4847916; doi:10.1371/journal.pone.0153565)
Supplement: S1 Table — Where MRCA is the most recent common ancestor of the two taxa that span the clade being dated on the tree. (DOCX) [file pone.0153565.s001.docx]

**S1. Table 1.** **Summary of nine Orders, dates used and the reference pertaining to each (Shapcott *et al*., 2015) used to date the SEQ rainforest phylogenetic tree in the PATHd8 program. Where MRCA is the most recent common ancestor of the two taxa that span the clade being dated on the tree.**

| Order | Minimum Age Constraint (ma) | Node defined as MRCA | Reference |  |
| --- | --- | --- | --- | --- |
| **Magnoliales** | 112 | *Euphrasia bella* & *Galbulimima baccata* | Mohr, B. & Bernardes de Oliveira, M. (2004) |  |
| **Sapindales** | 55.8 | *Cossinia australiana* & *Toechima dasyrrhache* | Manchester, S.R. (2001) |  |
| **Ericales** | 89.3 | *Acrothamnus spathaceus* & *Planchonia careya* | Nixon, K.C. & Crepet, W.L. (1993) |  |
| **Fabales** | 90.3 | *Barklya syringifolia* & *Guilfoylia monostylis* | Koenen, E. *et al.,* (2013) |  |
| **Myrtales** | 65 | *Lophostemon suaveolens* & *Melaleuca bracteata* | Crepet, W.L. *et al.,* (2004) |  |
| **Malvales** | 33.9 | *Commersonia bartramia* & *Grewia latifolia* | Manchester, S.R. (1999) |  |
| **Malpighiales** | 89.3 | *Croton acronychioides* & *Glochidion lobocarpum* | Crepet, W.L. & Nixon, K.C. (1998) |  |
| **Apiales** | 37.2 | *Bursaria incana* & *Mackinlaya macrosciadea* | Manchester, S.R. (1999) |  |
| **Laurales** | 100 | *Neolitsea dealbata* & *Daphnandra tenuipes* | Massoni, J. *et al.,* (2015) |  |
