# Supplementary material for: Patterns of Phylogenetic Diversity of Subtropical Rainforest of the Great Sandy Region, Australia Indicate Long Term Climatic Refugia
Source: PLoS One. 2016 Apr 27;11(4):e0153565. doi: 10.1371/journal.pone.0153565 (PMC4847916; doi:10.1371/journal.pone.0153565)
Supplement: S2 Table — The species name, Genbank accession codes for each of the three DNA barcode markers used as well as the Queensland Herbarium (BRI) collector name or number which is used by BRI as specimen identifier as well as the BRI accession number where available at time of submission. (DOCX) [file pone.0153565.s002.docx]

| Species | *matK* Accession | *trnH-psbA* Accession | *rbcLa* Accession | Collector Number | Queensland Herbarium  (BRI) Accession |
| --- | --- | --- | --- | --- | --- |
| *Acmena hemilampra* subsp*. hemilampra* | KU564534 | KU564629 | KU564749 | P.I.Forster PIF3952 | AQ835636 |
| *Acronychia littoralis* | KU564535 | KU564630 | KU564750 | P.I.Forster |  |
| *Acrothamnus spathaceus* | KU564536 | KU564631 | KU564751 | G.Leiper | AQ836002 |
| *Actephila sessilifolia* |  |  | KU564752 | P.I.Forster PIF30482 |  |
| *Alectryon pubescens* | KU564537 | KU564632 | KU564753 | P.I.Forster PIF39071 | AQ835194 |
| *Alectryon subcinereus* | KU564538 | KU564633 | KU564754 | P.I.Forster PIF40552 | AQ836052 |
| *Alphitonia oblata* | KU564539 | KU564634 | KU564755 | M.G.Howard MGH001 |  |
| *Ancistrachne uncinulata* |  | KU564635 | KU564756 | P.I.Forster PIF39150 | AQ835269 |
| *Aneilema acuminatum* |  |  | KU564757 | P.I.Forster PIF39070 | AQ835193 |
| *Antirhea putaminosa* | KU564540 | KU564636 | KU564758 | M.G.Howard MGH047 |  |
| *Archidendron hendersonii* |  | KU564637 | KU564759 | P.I.Forster PIF40508 | AQ836041 |
| *Archidendron lovelliae* | KU564541 | KU564638 | KU564760 | P.I.Forster PIF39525 | AQ835639 |
| *Atalaya calcicola* | KU564542 | KU564639 | KU564761 | M.G.Howard MGH054 |  |
| *Atalaya collina* | KU564543 | KU564640 | KU564762 | W.J.McDonald |  |
| *Australina pusilla* subsp. *pusilla* | KU564544 | KU564641 | KU564763 | P.I.Forster PIF40553 | AQ836053 |
| *Austrostipa ramossima* | KU564545 | KU564642 | KU564764 | P.I.Forster PIF39776 | AQ835186 |
| *Austrostipa verticillata* | KU564546 | KU564643 | KU564765 | P.I.Forster PIF39065 |  |
| *Backhousia* sp *Beenleigh* | KU564547 | KU564644 | KU564766 | G.Leiper Weber102 | AQ832193 |
| *Backhousia* sp *Belgama* | KU564548 | KU564645 | KU564767 | W.J.McDonald |  |
| *Callitris columellaris* |  | KU564646 | KU564768 | M.G.Howard MGH002 |  |
| *Callitris rhomboidea* |  | KU564647 | KU564769 | P.I.Forster PIF40558 | AQ836058 |
| *Canavalia papuana* | KU564549 | KU564648 | KU564770 | M.G.Howard MGH031 |  |
| *Capparis loranthifolia* var. *bancroftii* | KU564550 |  |  | P.I.Forster PIF40629 | AQ836360 |
| *Capparis mitchellii* | KU564551 | KU564649 | KU564771 | P.I.Forster PIF40653 | AQ836374 |
| *Cassinia compacta* | KU564552 | KU564650 | KU564772 | P.I.Forster PIF40546 |  |
| *Cayratia japonica* |  | KU564651 | KU564773 | M.G.Howard MGH024 |  |
| *Cayratia saponaria* | KU564553 | KU564652 | KU564774 | P.I.Forster PIF39162 | AQ835281 |
| *Cinnamomum baileyanum* | KU564554 | KU564653 | KU564775 | P.I.Forster PIF39535 | AQ835649 |
| *Cleistanthus dallachyanus* |  | KU564654 | KU564777 | N.D.Hoy s.n. | AQ836067 |
| *Clematis aristata* | KU564556 | KU564655 | KU564778 | L.Weber s.n. | AQ836095 |
| *Clematis fawcettii* | KU564557 |  | KU564779 | G.Leiper s.n. | AQ836006 |
| *Clerodendrum longiflorum* var. *glabrum* | KU564558 | KU564656 | KU564780 | A.Shapcott MGH055 |  |
| *Codonocarpus attenuatus* |  | KU564657 |  | P.I.Forster PIF40589 | AQ836131 |
| *Codonocarpus attenuatus* |  |  | KU564781 | B.Jeffers s.n. | AQ836257 |
| *Commelina diffusa* |  |  | KU564782 | P.I.Forster PIF39112 | AQ835232 |
| *Corchorus cunninghamii* | KU564559 | KU564658 | KU564783 | G.Guymer D62 |  |
| *Cordia dichotoma* | KU564560 | KU564659 | KU564784 | A.Shapcott MGH046 |  |
| *Cordyline stricta* | KU564561 | KU564660 | KU564785 | P.I.Forster PIF40531 | AQ836066 |
| *Cryptocarya vulgaris* | KU564563 | KU564662 | KU564787 | W.J.McDonald |  |
| *Cyperus rupicole* |  | KU564663 | KU564788 | P.I.Forster |  |
| *Daphnandra apetala* | KU564565 | KU564665 | KU564789 | P.I.Forster PIF40491 | AQ836022 |
| *Dendrocnide moroides* | KU564566 | KU564666 | KU564790 | S.Shaw |  |
| *Denhamia cunninghamii* | KU564567 |  |  | P.I.Forster PIF40665 | AQ836382 |
| *Denhamia oleaster* | KU564568 | KU564667 | KU564791 | M.B.Thomas | AQ489021 |
| *Dioscorea transversa* |  | KU564668 | KU564792 | M.G.Howard MGH004 |  |
| *Ehretia grahamii* | KU564569 | KU564669 | KU564793 | P.I.Forster |  |
| *Endiandra globosa* | KU564571 | KU564671 | KU564795 | P.I.Forster PIF40493 | AQ836023 |
| *Endiandra globosa* | KU564572 |  |  | S.Shaw |  |
| *Euphrasia bella* | KU564573 | KU564672 | KU564796 | P.Box s.n. | AQ836089 |
| *Eupomatia bennettii* | KU564574 | KU564673 | KU564797 | P.I.Forster PIF40527 | AQ836018 |
| *Flueggea leucopyrus* | KU564575 | KU564674 | KU564798 | M.G.Howard MGH034 |  |
| *Fontainea australis* |  |  | KU564799 | P.I.Forster PIF40500 | AQ836035 |
| *Freycinetia excelsa* | KU564576 |  | KU564800 | G.Leiper s.n. | AQ836061 |
| *Freycinetia scandens* | KU564577 |  | KU564801 | G.Leiper s.n. | AQ836062 |
| *Gaultheria viridicarpa* | KU564578 | KU564675 | KU564802 | P.Box s.n. | AQ836088 |
| *Geniostoma rupestre* var. *australianum* | KU564579 |  | KU564803 | M.G.Howard MGH053 |  |
| *Glycosmis trifoliata* |  | KU564676 | KU564806 | W.J.McDonald |  |
| *Gossia hillii* |  |  | KU564807 | P.I.Forster PIF40585 | AQ836084 |
| *Gymnanthera oblonga* | KU564582 | KU564677 | KU564808 | P.I.Forster PIF40083 | AQ836439 |
| *Gynothochodes umbellata* | KU564583 | KU564678 | KU564809 | P.I.Forster PIF39520 | AQ835634 |
| *Heterostemma acuminatum* | KU564584 |  |  | M.G.Howard MGH037 |  |
| *Heterostemma acuminatum* |  |  | KU564810 | M.G.Howard MGH044 |  |
| *Jasminum simplicifolium* subsp. *australiense* |  |  | KU564811 | P.I.Forster PIF40609 | AQ836199 |
| *Lenwebbia prominens* |  |  | KU564812 | P.I.Forster PIF40529 | AQ836015 |
| *Lenwebbia* sp Blackall Range (P.R. Sharpe 5387) | KU564585 | KU564679 | KU564813 | G.Leiper s.n. | AQ836212 |
| *Lenwebbia* sp Main Range (P.R. Sharpe 4877) | KU564586 | KU564680 | KU564814 | P.I.Forster PIF15694 | AQ631257 |
| *Leucopogon mitchellii* |  | KU564681 | KU564815 | P.I.Forster PIF40670 | AQ836391 |
| *Leucopogon* sp Lamington (G.Leiper AQ633386) | | KU564682 | KU564816 | P.I.Forster PIF40535 | AQ836065 |
| *Libertia paniculata* | KU564587 | KU564683 | KU564817 | P.I.Forster PIF40550 | AQ836046 |
| *Lycianthes shanesii* | KU564588 | KU564684 | KU564818 | M.G.Howard MGH040 |  |
| *Mallotus discolor* |  | KU564685 | KU564819 | W.J.McDonald |  |
| *Mallotus repandus* |  |  | KU564820 | M.G.Howard MGH052 |  |
| *Marsdenia glandulifera* |  |  | KU564821 | M.G.Howard MGH057 |  |
| *Marsdenia glandulifera* | KU564589 | KU564686 |  | M.G.Howard MGH058 |  |
| *Marsdenia Iloydii psba* |  | KU564687 |  | P.I.Forster PIF40625 | AQ836351 |
| *Marsdenia micolepis* | KU564590 | KU564688 | KU564822 | P.I.Forster PIF40643 | AQ836359 |
| *Marsdenia rostrata* | KU564591 | KU564689 | KU564823 | P.I.Forster PIF40549 |  |
| *Marsdenia viridiflora* subsp. *viridiflora* | KU564592 | KU564690 | KU564824 | P.I.Forster PIF39299 | AQ835420 |
| *Medicosma forsteri* | KU564593 | KU564691 | KU564825 | M.G.Howard MGH051 |  |
| *Melodinus acutiflorus* | KU564595 | KU564692 | KU564826 | P.I.Forster PIF40494 | AQ836026 |
| *Mucuna gigantea* |  |  | KU564828 | P.I.Forster PIF40520 | AQ835983 |
| *Mucuna gigantea* | KU564596 | KU564693 |  | M.G.Howard MGH006 |  |
| *Muihlenbeckia gracillima* |  | KU564694 | KU564829 | G.Leiper s.n. | AQ836134 |
| *Myrsine arenaria* |  | KU564695 | KU564830 | M.G.Howard MGH027 |  |
| *Myrsine crassifolia* | KU564597 | KU564696 | KU564831 | M.G.Howard MGH038 |  |
| *Neisosperma poweri* | KU564598 | KU564697 | KU564832 | S.Shaw |  |
| *Niemeyera prunifera* |  |  | KU564833 | W.J.McDonald |  |
| *Notelaea johnsonii* |  | KU564698 | KU564834 | P.I.Forster PIF40526 | AQ836019 |
| *Notelaea* sp. Indet |  | KU564699 | KU564835 | P.I.Forster PIF40528 | AQ836017 |
| *Notelaea venosa* | KU564599 |  | KU564836 | P.I.Forster PIF40551 | AQ836051 |
| *Olearia elliptica* | KU564601 | KU564700 |  | P.I.Forster PIF40536 | AQ836011 |
| *Oplismenus aemulus* |  | KU564701 | KU564838 | P.I.Forster PIF39110 | AQ835230 |
| *Oplismenus imbecillus* |  | KU564702 | KU564839 | P.I.Forster PIF39153 | AQ835272 |
| *Oplismenus mollis* |  | KU564703 | KU564840 | P.I.Forster PIF40517 | AQ835985 |
| *Owenia cepiodora* | KU564602 | KU564704 |  | P.I.Forster PIF34140 |  |
| *Palmeria racemosa* |  | KU564705 | KU564841 | M.G.Howard MGH019 |  |
| *Panicum pygmaeum* |  | KU564706 | KU564842 | G.Leiper s.n. | AQ 836133 |
| *Parsonsia induplicata* |  | KU564707 | KU564843 | P.I.Forster PIF40525 | AQ836020 |
| *Phyllanthus similis* |  | KU564708 | KU564844 | M.G.Howard MGH032 |  |
| *Pimelea linifolia* subsp. *linifolia* |  | KU564709 | KU564845 | M.G.Howard MGH090 |  |
| *Pipturus argenteus* | KU564603 | KU564710 | KU564846 | W.J.McDonald |  |
| *Pisonia aculeata* | KU564604 | KU564711 | KU564847 | M.G.Howard MGH041 |  |
| *Pitaviaster haplophyllus* |  | KU564712 | KU564848 | P.I.Forster PIF 29075 |  |
| *Plectranthus nitidus* |  | KU564713 | KU564849 | P.I.Forster PIF40576 | AQ836076 |
| *Polyscias australiana* |  | KU564714 | KU564850 | M.G.HowardMGH021 |  |
| *Pomaderris notata* |  | KU564716 | KU564852 | G.Leiper s.n. | AQ836003 |
| *Proiphys cunninghamii* |  | KU564717 | KU564853 | P.I.Forster PIF39152 | AQ835271 |
| *Prostanthera ovalifolia* | KU564606 | KU564718 | KU564854 | P.I.Forster PIF40534 | AQ836012 |
| *Pseuderanthemum variabile* |  | KU564719 | KU564855 | P.I.Forster PIF39066 | AQ835187 |
| *Psydrax odorata forma (*Bridgooda P.I.Forster PIF5657) | KU64608 |  | KU564857 | P.I.Forster PIF40654 | AQ836371 |
| *Randia moorei* | KU564609 | KU564721 | KU564858 | P.I.Forster PIF40506 | AQ836027 |
| *Rhagodia parabolica* | KU564610 | KU564722 | KU564859 | P.I.Forster PIF39410 | AQ835528 |
| *Rhodamnia angustifolia* |  | KU564723 | KU564860 | W.J.McDonald |  |
| *Rhodamnia spongiosa* |  | KU564724 | KU564861 | W.J.McDonald |  |
| *Rhynchosia acuminatissima* | KU564611 |  |  | M.G.Howard MGH049 |  |
| *Rhynchosia acuminatissima* |  | KU564725 | KU564862 | M.G.Howard MGH043 |  |
| *Ripogonum brevifolium* |  | KU564726 | KU564863 | P.I.Forster PIF39069 | AQ835191 |
| *Sambucus gaudichaudiana* | KU564612 | KU564727 | KU564864 | M.G.Howard MGH018 |  |
| *Senna gaudichaudii* |  | KU564728 |  | M.G.HowardMGH036 |  |
| *Sicyos australis* |  | KU564729 |  | P.I.Forster PIF40738 |  |
| *Sloanea macbrydei* |  | KU564730 | KU564865 | W.J.McDonald |  |
| *Solanum aviculare* | KU564614 | KU564731 | KU564866 | P.I.Forster PIF40557 | AQ836057 |
| *Solanum furfuraceum* | KU564616 | KU564733 | KU564868 | M.G.Howard MGH045 |  |
| *Solanum inaequilaterum* |  | KU564734 | KU564869 | G.Leiper s.n. | AQ836005 |
| *Solanum opacum* | KU564617 | KU564735 | KU564870 | P.I.Forster PIF40548 | AQ836049 |
| *Solanum ditrichum* | KU564615 | KU564732 | KU564867 | G.Leiper | AQ836211 |
| *Solanum rixosum* | KU564618 | KU564736 | KU564871 | P.I.Forster PIF40753 |  |
| *Solanum serpens* | KU564619 |  | KU564872 | G.Leiper s.n. | AQ836004 |
| *Solanum stupefactum* |  | KU564737 | KU564873 | P.I.Forster PIF40626 | AQ836190 |
| *Sophora fraseri* | KU564620 | KU564738 | KU564874 | W.J.McDonald |  |
| *Sophora tomentosa* subsp. *australis* | KU564621 | KU564739 | KU564875 | W.J.McDonald |  |
| *Stephania renifolia* | KU564622 | KU564740 | KU564876 | P.I.Forster PIF39545 | AQ835659 |
| *Stictocardia tiliifolia* | KU564623 | KU564741 | KU564877 | P.I.Forster PIF40510 | AQ835994 |
| *Symplocos thwaitesii* | KU564624 | KU564742 | KU564878 | G.Leiper s.n. | AQ836094 |
| *Syzygium johnsonii* | KU564626 | KU564744 | KU564880 | M.G.Howard MGH022 |  |
| *Syzygium moorei* |  |  | KU564881 | P.I.Forster PIF40497 | AQ836037 |
| *Tapeinosperma pseudojambosa* |  | KU564745 | KU564882 | W.J.McDonald |  |
| *Tylophora grandiflora* |  | KU564746 | KU564884 | P.I.Forster PIF39151 | AQ835270 |
| *Xylosma ovatum* |  | KU564747 | KU564885 | W.J.McDonald |  |
| *Zieria southwellii* |  | KU564748 | KU564886 | G.Leiper s.n. | AQ836001 |
